# Post-Synthetic Reduction of Pectin Methylesterification Causes Morphological Abnormalities and Alterations to Stress Response in *Arabidopsis thaliana*

**DOI:** 10.3390/plants9111558

**Published:** 2020-11-12

**Authors:** Nathan T. Reem, Lauran Chambers, Ning Zhang, Siti Farah Abdullah, Yintong Chen, Guanhua Feng, Song Gao, Junmarie Soto-Burgos, Gennady Pogorelko, Diane C. Bassham, Charles T. Anderson, Justin W. Walley, Olga A. Zabotina

**Affiliations:** 1 Department of Biochemistry, Biophysics, and Molecular Biology, Iowa State University, Ames, IA 50011, USA; ntr27@cornell.edu (N.T.R.); 72lauran@gmail.com (L.C.); ningz@iastate.edu (N.Z.); sitifarahabdullah@yahoo.com (S.F.A.); gennady@iastate.edu (G.P.); 2 Department of Biology, The Pennsylvania State University, University Park, PA 16802, USA; yuc53@psu.edu (Y.C.); guanhuafengkiki@outlook.com (G.F.); cta3@psu.edu (C.T.A.); 3 Department of Plant Pathology & Microbiology, Iowa State University, Ames, IA 50011, USA; sgy0097@iastate.edu (S.G.); jwalley@iastate.edu (J.W.W.); 4 Department of Genetics, Development & Cell Biology, Iowa State University, Ames, IA 50011, USA; junmarie@iastate.edu (J.S.-B.); bassham@iastate.edu (D.C.B.)

**Keywords:** *Arabidopsis thaliana*, pectin methylesterification, cell wall signaling, *Botrytis cinerea*, pattern-triggered immunity

## Abstract

Pectin is a critical component of the plant cell wall, supporting wall biomechanics and contributing to cell wall signaling in response to stress. The plant cell carefully regulates pectin methylesterification with endogenous pectin methylesterases (PMEs) and their inhibitors (PMEIs) to promote growth and protect against pathogens. We expressed *Aspergillus nidulans* pectin methylesterase (AnPME) in *Arabidopsis thaliana* plants to determine the impacts of methylesterification status on pectin function. Plants expressing AnPME had a roughly 50% reduction in methylester content compared with control plants. AnPME plants displayed a severe dwarf phenotype, including small, bushy rosettes and shorter roots. This phenotype was caused by a reduction in cell elongation. Cell wall composition was altered in AnPME plants, with significantly more arabinose and significantly less galacturonic acid, suggesting that plants actively monitor and compensate for altered pectin content. Cell walls of AnPME plants were more readily degraded by polygalacturonase (PG) alone but were less susceptible to treatment with a mixture of PG and PME. AnPME plants were insensitive to osmotic stress, and their susceptibility to *Botrytis cinerea* was comparable to wild type plants despite their compromised cell walls. This is likely due to upregulated expression of defense response genes observed in AnPME plants. These results demonstrate the importance of pectin in both normal growth and development, and in response to biotic and abiotic stresses.

## 1. Introduction

The plant cell wall is an important, complex component of all plant cells. Changes in the wall composition and structure can affect a plant’s fitness and response to stresses. The cell wall is composed primarily of polysaccharides, as well as lignin, proteins, and ions that associate with or bind to the polysaccharides [1]. The polysaccharides of the primary cell wall make up the bulk of its dry weight and are divided into three categories: cellulose, hemicelluloses, and pectins [2]. Pectins constitute 20–35% of the primary cell wall, and also reside in the middle lamellae between cells [3]. Pectins are acidic heteropolymers of three distinct types containing galacturonic acid (GalA). Homogalacturonan (HG) is composed of unbranched α-1,4-linked GalA residues that are gradually demethylesterified as the plant ages [4,5]. Rhamnogalacturonan I (RG-I) is composed of alternating α-1,4-GalA and α-1,2-Rhamnose (Rha) residues with sidechains of varying composition [6], and rhamnogalacturonan II (RG-II) is composed of an α-1,4-linked GalA backbone with complex sidechains [6,7,8]. Pectins exist as continuous heteropolymers in muro with long stretches of HG interspersed between shorter stretches of RG-I and RG-II [9].

Plant cell wall proteins (CWP) play important roles in various abiotic and biotic stress responses, and some CWPs were found to be key factors in adaptation to many stresses [10]. Signaling pathways for both abiotic and biotic stresses reveal intersections between responses and share regulatory principles [11]. Another plant cell wall component, lignin, is deeply involved in control of plant growth and immunity functioning. Remodeling of cell walls in transgenic plants with altered lignin content was shown to also result in release of pectic oligosaccharide elicitors and defensive PR gene expression [12].

Pectin plays an important role in plant and organ morphology: the degree of pectin methylesterification affects cell wall biomechanics via Ca^2+^-crosslinking, and consequently affects organ formation [13]. Pectin is critical for cell wall porosity and elasticity, protection against degradation, and in plant immunity as a source of signaling molecules released upon stress and acting as triggers for pattern-triggered immunity [14,15,16]. These functions are determined and tuned by the interplay between pectin methylesterases (PME) and pectin methylesterase inhibitors (PMEI) [17]. Arabidopsis plants expressing catalytically inactive GalA biosynthetic enzymes (GAE1 and GAE6) have less cell wall pectin, brittle leaves, and higher susceptibility to *Botrytis cinerea* in comparison with wild type (WT) plants [18]. Overexpression of a seed-specific PME alters embryo morphology and reduces cell size in Arabidopsis [19]. Constitutive overexpression of an endogenous AtPMEI increases seed methylesterification and germination rates. AtPMEI overexpression also causes an unusual growth phenotype: stunted growth and thick, twisted stems, particularly at points where cauline leaves or flowers would normally separate from the main stem [20,21]. Overexpression of Arabidopsis PMEI1 and PMEI2 decreases cell adhesion and increases the efficiency of protoplast isolation due to decreased wall recalcitrance to enzyme degradation [22]. Pectin also contributes to guard cell mechanics. Arabinan is critical for guard cell closure, and plants defective in pectin-modifying genes such as PME6 or a polygalacturonase show inhibition of stomatal opening and closing [23,24,25]. Such mechanical changes in guard cells were shown to be caused by cell wall plasticity alteration via apoplastic Ca^2+^ modulation as a part of plant adaptation to heat stress [26].

PMEs are a large family of enzymes belonging to the CAZy class CE-8 (EC3.1.1.11) and are present in plants, fungi, and bacteria [27]. The Arabidopsis genome contains 66 distinct PMEs [28]. AtPME usually de-esterifies HG in a blockwise fashion; 9 or more consecutive demethylesterified GalA residues accommodate ionic Ca^2+^-mediated crosslinks between HG chains to rigidify the cell wall [15,29,30]. Fungal PMEs generally de-esterify HG in sporadic, non-blockwise fashion [31,32]. Fungal PMEs do not usually promote Ca^2+^-mediated crosslinks; rather, they enhance the efficiency of polygalacturonases and pectate lyases to facilitate pathogenesis [5,33].

The Arabidopsis genome contains 71 PME inhibitors (AtPMEIs), and some have been shown to regulate AtPMEs during plant development and during pathogenesis [28,34]. AtPMEIs largely inhibit plant PMEs; most do not inhibit fungal PMEs due to their structure [35,36]. However, recently it has been demonstrated that several AtPMEIs are critical for cell wall integrity (CWI) during pathogenesis. After *B. cinerea* inoculation, transcripts of AtPMEI10, AtPMEI11, AtPMEI12 are upregulated, and knockout mutants of these genes displayed increased pathogen susceptibility [37].

Pathogens must contend with the plant cell wall before successfully colonizing the plant. Both biotrophic and necrotrophic pathogens secrete cell wall degrading enzymes (CWDEs), which macerate the wall matrix and allow access to the cytoplasm [38]. Pectin-degrading enzymes such as polygalacturonases, rhamnogalacturonan hydrolases, pectin lyases, and pectate lyases are among the first CWDEs secreted by pathogenic fungi [39,40,41,42]. CWDE expression is induced in fungi during pathogenesis, and is correlated with their virulence [43,44,45,46]. Endogenous CWDEs are important during pathogenesis, too. For example, expression of AtPME17 is regulated by defense signaling pathways, and *atpme17* mutants exhibited increased susceptibility to *Botrytis cinerea*, suggesting its importance for early defense response [47]. Thus, polygalacturonases and PMEs are critical for pathogenesis [48,49].

The process of sensing byproducts of pectin degradation such as oligogalacturonides (OGs) is carried out by membrane-bound pattern-recognition receptors (PRRs). PRRs recognize OGs as damage-associated molecular patterns (DAMPs) and initiate defense responses [50,51]. For example, OGs are sensed by members of the WAK family of PRRs, which initiate signaling through a set of mitogen-activated protein kinases (MPKs), MPK6, and possibly MPK8, to initiate a defense response [52]. It has also been suggested that volatilized methanol released by PME is bioactive and initiates a defense response [53,54].

This study explores the role of pectin methylesterification in CWI and the impact of cell wall de-esterification on plant growth and stress response. *Aspergillus nidulans* pectin methylesterase (AnPME) was constitutively expressed under a 35S promoter and exported to the apoplast in *Arabidopsis thaliana* plants. This approach allows cells to fully synthesize their cell walls in the same manner as wild-type (WT) plants (in contrast to knockouts of biosynthetic enzymes), and the AnPME, which is constitutively expressed in all tissues, modifies the cell wall to a higher degree than the tightly regulated AtPMEs [55]. The transgenic plants showed a severe dwarf phenotype, reduced rate of growth, decreased cell size, and cell wall compositional changes. A general reduction in stomatal pore length was also observed in AnPME plants, though no change in guard cell function was detected. In addition, the AnPME plants were insensitive to salt and osmotic stress and upregulated expression of defense response genes as shown by qPCR analyses. Despite increased cell wall digestibility, AnPME plants did not show changes in susceptibility to the fungal necrotroph *B. cinerea* in comparison with wild type plants, most likely because of their constitutively upregulated defense responses.

## 2. Results

### 2.1. Arabidopsis Plants Expressing AnPME have a Dwarf Phenotype and Reduced Cell Expansion

**Figure 1:**
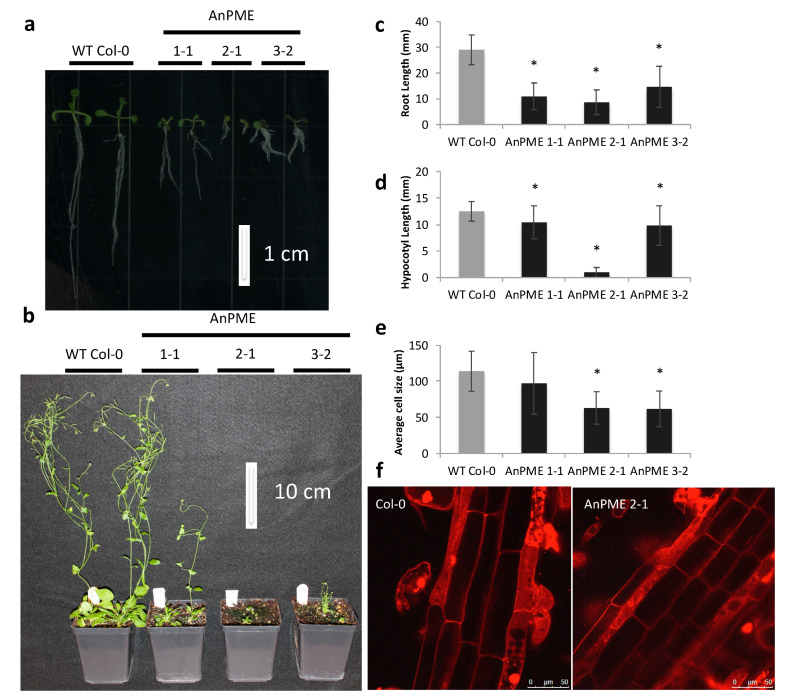
AnPME plants display a severe stunted phenotype. (**a**) Seven-day-old seedlings exhibit reduced root growth and leaf expansion; (**b**) five-week old AnPME plants with reduced leaf and rosette area, as well as shorter stems; (**c**) Root length is reduced in seven-day old AnPME plants (*n* = 60); (**d**) Length of hypocotyls is reduced in etiolated AnPME seedlings (*n* = 60); (**e**,**f**) Cell elongation is inhibited in AnPME plants determined through confocal imaging of propidium iodide stained root tissue (*n* ≥ 18 root cells from 6 plants). Error bars represent standard deviation. Asterisks indicate statistical significance (*p* < 0.05, student’s *t*-test).

Three independent homozygous transgenic events were selected for this study based on their varying degree of phenotype shown (AnPME 1-1, 2-1, and 3-2). All three lines of AnPME-expressing Arabidopsis plants exhibited impaired growth in almost all observed organs. Cotyledons of seven-day-old seedlings expanded normally, but plant growth was visibly reduced after emergence of the first leaves. AnPME plants displayed reduced rosette area, smaller leaves, siliques, flowers, and shorter stems (Figure 1a,b). Roots of AnPME plants were approximately 50% shorter in comparison with WT Col-0 (Figure 1c). Reduced hypocotyl length was also observed in etiolated seedlings (Figure 1d). Confocal imaging of seven-day-old seedling roots revealed a significant reduction in cell length in two independent transgenic AnPME lines (AnPME 2-1 and 3-2) relative to WT Col-0, whereas the cell size in AnPME 1-1 plants was not statistically different (Figure 1e,f).

**Figure 2:**
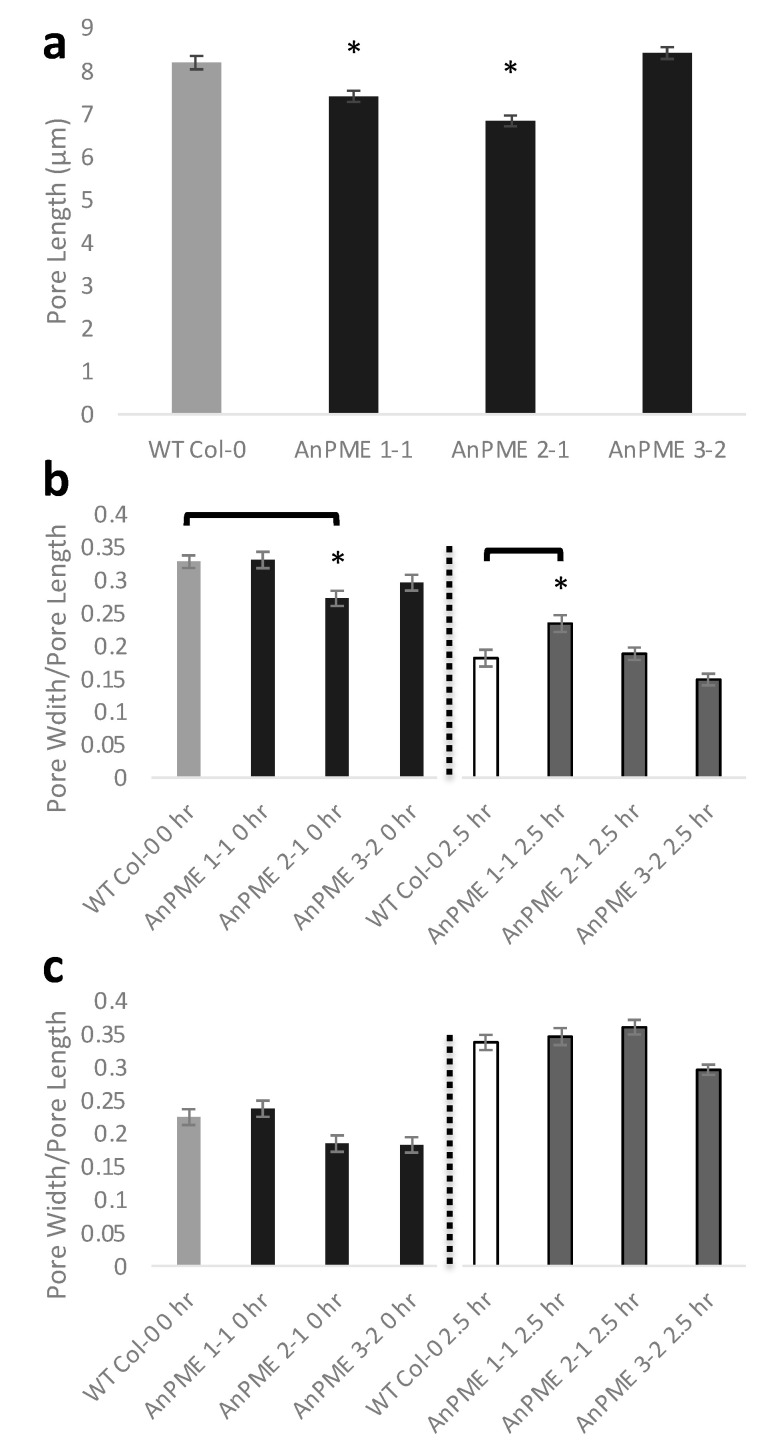
Six-day-old seedlings of AnPME lines show decreased stomatal size but have stomatal functions that are similar to WT. (**a**) Stomatal pore length in WT Col-0 and AnPME lines (*n* ≥ 542 stomatal complexes from six independent experiments); (**b** and **c**) Stomatal responses to dark (**b**) and light (**c**) treatment in WT Col-0 and AnPME lines, represented by pore width to pore length ratio (*n* ≥ 124 stomatal complexes from three independent experiments). Error bars represent standard deviation. Asterisks indicate statistical significance (*p* < 0.05, one-way ANOVA).

Guard cells of AnPME plants were observed to reveal whether stomatal development or function were impaired. Stomatal pore length in AnPME 1-1 and 2-1 were significantly reduced, although no changes were detected in AnPME 3-2 (Figure 2a). Stomatal responses to dark and light treatments were also studied, but little difference was seen in all three lines of AnPME plants. Before dark treatment, AnPME 2-1 exhibited a significantly lower length:width ratio, but no difference was observed in this line after dark treatment (Figure 2b). AnPME 1-1 displayed increased length:width after dark treatment; the other two AnPME lines did not (Figure 2b). No change was observed before and after light treatment (Figure 2c).

### 2.2. Cell Walls in AnPME Plants Have Reduced Methylester Content and Modified Recalcitrance to CWDEs

**Figure 3:**
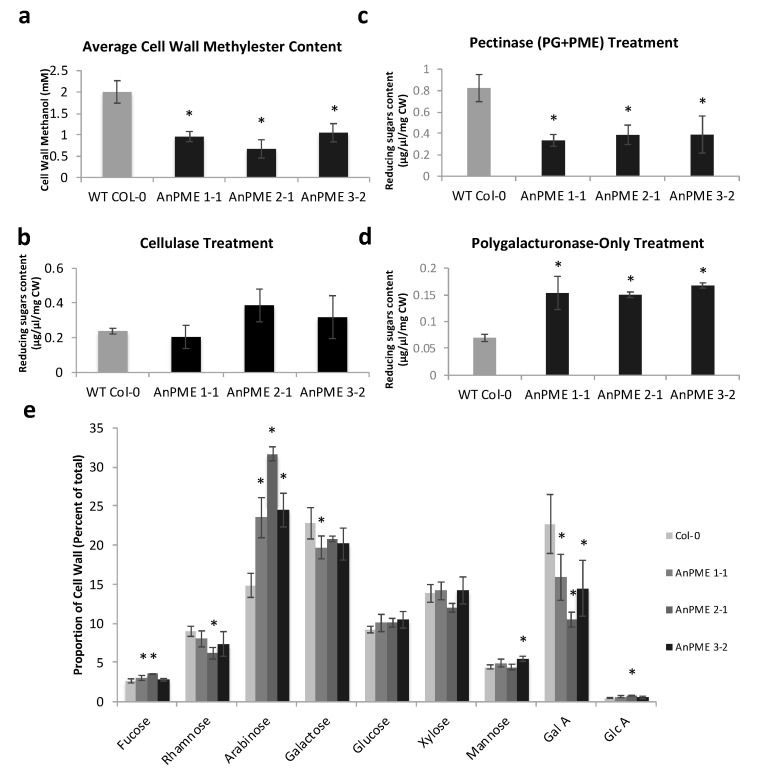
CW characterization and comparison between Col-0 and AnPME. (**a**) Methylester content in WT Col-0 and AnPME cell walls; (**b**) Amount of reducing sugars released into solution after treatment of cell wall with cellulase/cellobiase solution; (**c**) Amount of reducing sugars released into solution after treatment of cell wall with polygalacturonase/pectin methylesterase solution; (**d**) Amount of reducing sugars released into solution after treatment of cell wall with polygalacturonase; (**e**) Monosaccharide composition of cell walls. Error bars represent standard deviation. Asterisks indicate statistical significance (*p* < 0.05, one-way ANOVA; *n* = 3 biological replicates of 10 pooled plants each).

Cell wall methylester content in AnPME plants was reduced by 30–50% in comparison with WT Col-0 cell walls (Figure 3a). To assess the impact of reduced methylesterification on cell wall degradability, the transgenic and wild type cell walls were digested with cellulases. Amount of reducing sugars released after cellulase treatment was not significantly different between AnPME plants and WT Col-0 (Figure 3b). When cell walls from AnPME and WT plants were treated with a pectinase cocktail (polygalacturonase + pectin methylesterase), a lower amount of reducing sugars was released from AnPME cell walls than from WT cell wall (Figure 3c). However, when the cell walls were treated with polygalacturonase alone, the amount of reducing sugars released from AnPME cell walls was significantly higher in comparison with WT (Figure 3d). Compositional analysis revealed that cell walls of all three AnPME lines had reduced galacturonic acid and increased arabinose content relative to WT. Significant differences between at least one AnPME line and WT were also observed in fucose, rhamnose, galactose, mannose, and glucuronic acid (Figure 3e).

### 2.3. Plants Expressing AnPME Display Decreased Sensitivity to Salt and Osmotic Stresses and Increased Expression of Defense Response Genes

**Figure 4:**
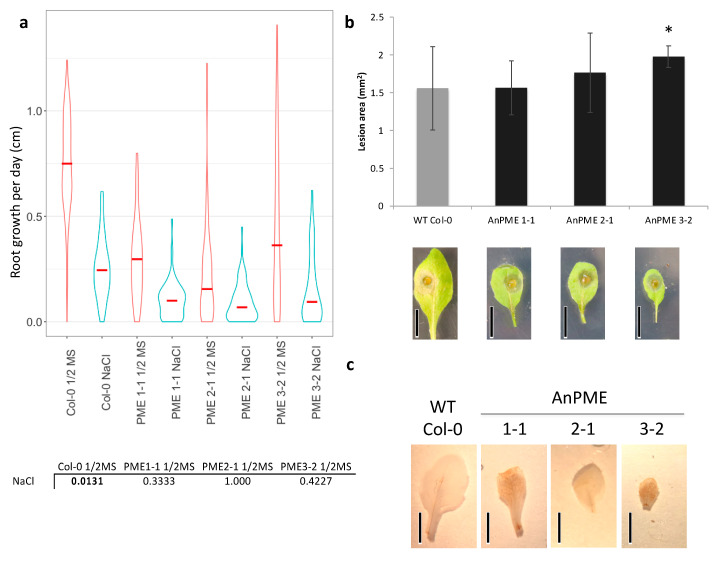
Plant response to stresses. (**a**) Root growth comparison between plants grown in optimal conditions (1/2MS) and salt stress (100 mM NaCl). Red lines indicate median values. Below box plot, adjusted *p*-values (Bonferroni) comparing the same plants grown on 1/2MS and salt. Bold values indicate statistical significance (*p* < 0.05, ANOVA; *n* ≥ 75); (**b**) Lesion areas 48 h after inoculation with *B. cinerea* (*n* = 12); (**c**) Uninoculated rosette leaves stained with 3,3′-diaminobenzine showing ROS accumulation in AnPME plants. Error bars represent standard deviation. Asterisks indicate statistical significance (*p* < 0.05, student’s *t*-test). Scale bars = 1 cm.

To investigate whether reduced pectin methylesterification impacts plant stress responses, AnPME and WT Col-0 plants were subjected to salt and osmotic stress. Rates of root growth in plants grown on medium in the presence of stress (100 mM NaCl or 300 mM mannitol) and absence of stress (1/2MS; ½-strength Murashige and Skoog medium) were compared. While WT Col-0 root growth was significantly inhibited under salt stress conditions, AnPME root length was not significantly different compared with AnPME roots grown on salt-free medium (Figure 4a). The same pattern was observed comparing root growth in unstressed and mannitol-grown plants: WT Col-0 plants grown under osmotic stress showed a significant reduction in root growth, while roots of AnPME plants grown on mannitol-containing medium were not significantly different from their unstressed counterparts (Figure S1).

Because pectins are often demethylesterified by fungal pathogens during infection, we sought to determine the impact of cell wall demethylesterification on plant resistance to fungal necrotrophs. Four-week-old WT Col-0 and AnPME-expressing plants were challenged with *B. cinerea*, and lesion areas were measured 48 h after inoculation. No significant difference was detected in lesion area between WT Col-0 and AnPME plants 1-1 and 2-1. Lesion areas on AnPME 3-2 plants were significantly larger than WT (Figure 4b).

**Figure 5:**
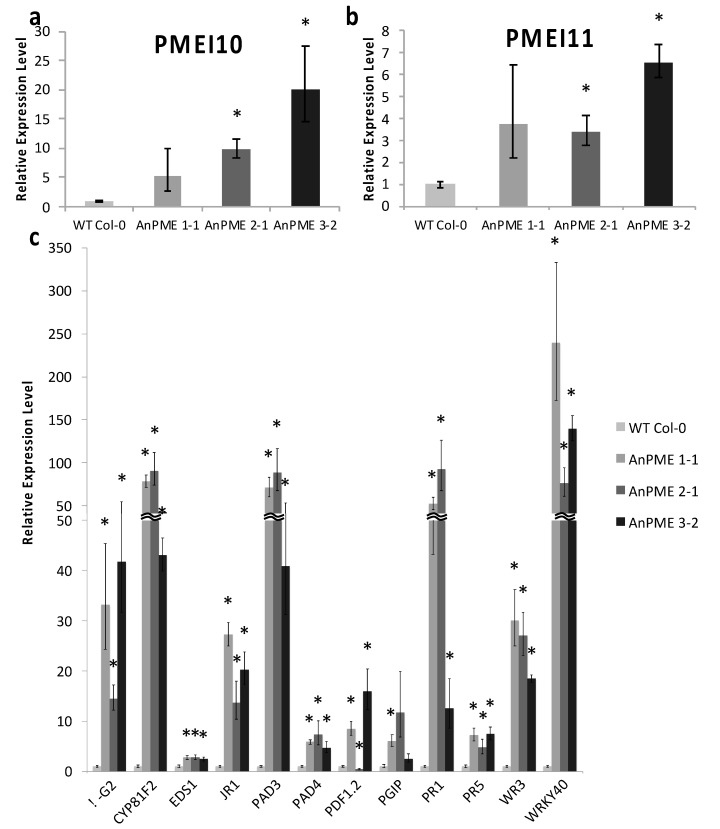
RT-qPCR of defense response genes. (**a**,**b**) Expression of PMEI10 and PMEI11 in plants grown under normal conditions (*n* = 5); (**c**) Expression of defense response genes grown under normal conditions (*n* = 5). Error bars represent standard deviation. Asterisks indicate statistical significance (*p* < 0.05, one-way ANOVA).

It is common for plants to initiate defense pathways in response to changes in the cell wall. One of the earliest responses to pathogenesis is reactive oxygen species (ROS) accumulation, in particular H_2_O_2_. Rosette leaves were incubated with 3,3′-diaminobenzene to stain H_2_O_2_, which revealed an increased ROS accumulation in AnPME plants (Figure 4c). To determine which defense responses were being activated in AnPME plants, RNA was extracted from sterile-grown plants, and an array of defense response genes were assayed. Interestingly, sterile-grown AnPME 2-1 and 3-2 plants upregulated expression of PMEI10 and PMEI11, two PMEIs known to be induced under pathogenesis by *B. cinerea* (Figure 5a,b). Defense response gene expression was also upregulated (Figure 5c). This includes β-Glucanase2 (β-G2), Cytochrome P450 81F2 (CYP81F2), Enhanced Disease Susceptibility 1 (EDS1), Jasmonate Responsive 1 (JR1), Phytoalexin-Deficient 3 (PAD3), Phytoalexin-Deficient 4 (PAD4), Plant Defensin 1.2 (PDF1.2), Pathogenesis Responsive 1 (PR1), Pathogenesis Responsive 5 (PR5), Wound Responsive 3 (WR3), and WRKY40 (Figure 5c). Polygalacturonase-Inhibiting Protein (PGIP) was upregulated in AnPME1-1, but was not significantly different in AnPME 2-1 or 3-2.

## 3. Discussion

Pectin is critical for plant development and response to stresses, including cell wall mechanical strength, cell-cell adhesion, and defense signaling [2,22,56]. However, due to the complexities of cell wall structure and signaling, it is difficult to pinpoint how pectin exerts control over these functions. Previous research has shown that constitutive expression of microbial hydrolases in the plant apoplast is a useful approach to investigate the impacts of cell wall post-synthetic modification on cell wall signaling, plant fitness, and stress responses [15,57,58,59,60,61]. Since plant growth is often halted under stress, this approach provides an opportunity to understand pectin’s contribution to both plant cell wall mechanics and plant response to stresses. This study has demonstrated that expression of fungal AnPME elicits defense responses in Arabidopsis and causes a dwarfed morphology, most likely due to pectin demethylesterification.

### 3.1. Microbial PME Expression Achieves Similar Phenotypes as Those Caused by Changes in Endogenous PME Activity

The AnPME enzyme expressed in Arabidopsis caused up to 50% reduction of pectin methylesterification, resulting in a severe dwarf phenotype most likely due to suppressed cell elongation (Figure 1a,e and Figure 3a). Several previous studies have observed a similar negative impact of reduced HG methylesterification on plant growth, in which pectin was altered either through knockout of endogenous PME [14], overexpression of PMEI [21,62,63], or knockout of the putative methyltransferases QUASIMODO2/TUMOROUS SHOOT DEVELOPMENT2 (QUA2/TSD2), COTTON GOLGI RELATED2 (CGR2), and CGR3 [64,65]. *qua2*/*tsd2* knockouts exhibited reduction in pectic HG biosynthesis and in cell elongation [65]. *cgr2 cgr3* double knockouts show reduced levels of pectin methylesterification and severe growth defects. The authors proposed that reduced HG methylesterification could increase Ca^2+^-dependent cross-linking, which limited cell wall expansion [64]. The AnPME plants in this study also most likely possess less expandable cell walls, resulting in reduced cell elongation (Figure 1e,f and Figure 2). This suggests that AnPME functions much like AtPME when expressed constitutively.

The non-blockwise de-esterification of HG by microbial PMEs promotes degradation of the cell wall by increasing accessibility of HG to the action of PGs [5,31,32,33]. Indeed, the cell walls from AnPME plants released significantly more reducing sugars than those of WT Col-0 plants when treated with polygalacturonase, confirming that AnPME makes the wall more accessible to PG degradation (Figure 3d). It is worth noting the results of cell walls treated with either PG+PME or PG alone. A comparison of reducing sugars released between the two pectin-degrading treatments (Figure 3c,d) shows that WT Col-0 cell walls with PG-only treatment yielded only 8% of the sugars yielded by WT cell walls treated with PG+PME. On the other hand, AnPME cell walls treated with PG-only yielded a much larger amount, between 38–45% of the total sugar content observed in AnPME cell walls treated with PG+PME (Figure 3c,d). This demonstrates that AnPME plants have significantly less HG available for additional de-esterification by added PME enzyme because a significant amount of HG was already de-esterified by the ectopically expressed PME. Likewise, treatment with PG alone yielded a significantly higher amount of reducing sugars in AnPME plants because de-esterified HG was readily available for PG digestion. Taken together, this suggests that transgenic expression of PME hastens wall degradation by facilitating hydrolysis by endogenous PG.

Interestingly, the cell walls from AnPME plants contained significantly less GalA and a higher amount of Ara, indicating that the ratio between HG and RG-1 is significantly altered (Figure 3e). This could occur due to the expressed PME increasing accessibility of HG to endogenous PGs, resulting in reduced levels of HG and causing a dwarf phenotype similar to what was observed for Arabidopsis and tobacco plants expressing microbial PG [66]. Arabinose-containing side chains are known to be involved in pectin-cellulose interaction, which can negatively impact cell wall flexibility, and could explain reduction of cell elongation in AnPME plants [23,67,68]. It is also possible that either HG biosynthesis is downregulated, or RG-I/RG-II biosynthesis is upregulated in AnPME plants. Further detailed analysis will be required to address this question.

### 3.2. Degree of Pectin Methylesterification Affects Plant Resistance to Stresses

Cell wall integrity (CWI) is the detection of changes in the cell wall and the subsequent signaling and compensatory responses. This typically involves a growth-defense tradeoff controlled through cell signaling, including hormone crosstalk [69]. For example, the plasma membrane co-receptors FLS2 and BAK1 bind bacterial flagellin to induce expression of the microRNA mir393, which represses auxin gene expression [70,71,72]. Jasmonic acid, a defense compound produced in plants in response to necrotrophic pathogenesis or insect herbivory, suppresses the auxin efflux transporter PIN2 [73], but can also be suppressed by brassinosteroid presence [74]. Endogenous AtPMEs, partially controlled by jasmonic acid signaling, also contribute to pathogen response [47]. WAKs also control the growth-defense tradeoff: either by signaling for cell expansion through MAP Kinase 3 (MPK3), or by initiating defense response through MPK6/MPK8 [56,75,76,77]. Given this tradeoff between growth and defense, we sought to determine whether the morphological abnormalities in AnPME plants were caused by disruption of cell wall biomechanics, or whether initiation of defense response was also a factor in the AnPME phenotype.

While WT Col-0 root growth was greatly suppressed under salt stress, the AnPME roots did not exhibit a reduction in growth (Figure 4a). Because the receptor-like kinase FERONIA interacts with pectin and mediates salt stress response through Ca^2+^ modulation, this may suggest that pectin methylesterification status is a key determinant to FERONIA signaling [78]. Since the free carboxyl groups on pectates hold a negative charge, demethylesterified HG in AnPME plants could potentially sequester more Na^+^ ions than in WT plants, minimizing the effect of high salinity on cell growth [79]. However, the observation that AnPME plants also showed no reduction in root growth under osmotic stress suggests pectin methylesterification may affect resistance to osmotic stress rather than salt stress, or that salt-stressed plants were responding to salt-induced osmotic pressure rather than Na^+^ ions [80]. This resistance to osmotic stress could be due to increased Ca^2+^-mediated crosslinks strengthening HG and thus preventing reduction in cell size, increased availability of freed carboxyl groups to retain water, or may be a result of osmotic stress signals passing through the same MPK3/MPK6 pathways as the growth-defense WAK signaling pathways (Figure S1) [11,81]. However, without directly measuring expression in the specific salt- and osmotic-stress signaling pathways, we are unable to determine the stress to which AnPME plants possess resistance [82].

Fungi secrete CWDEs, including PME, to infiltrate plants and gain access to the cytoplasm [38]. The success of other pectin-degrading enzymes depends on pectin methylesterification status (Figure 3c,d). Considering that de-esterified cell wall is more degradable by microbial PG (Figure 3d), we expected increased susceptibility to *B. cinerea*. Although AnPME 3-2 lesion areas were larger than WT plants, the AnPME 1-1 and 2-1 plants showed no difference from WT (Figure 4b). This indicates that higher digestibility of cell walls in AnPME plants does not significantly compromise their response to fungal pathogens, and may suggest that AnPME overexpression mimics endogenous AtPME activity towards pathogenesis [47]. While these results could be a side effect of a stiffer cell wall due to Ca^2+^-mediated crosslinking, it is more likely that heightened defense is responsible for this phenotype (Figure 4b and Figure 5). ROS production in healthy AnPME plants supports this because both salt and fungal pathogenesis are known to induce H_2_O_2_ production in plants in response to these stresses (Figure 4c).

Upregulation of PMEI10 and PMEI11, as well as defense response markers, demonstrated that strong defense responses were initiated in all three AnPME lines (Figure 5). AtPMEI10, AtPMEI11, and AtPMEI12 were previously identified as upregulated in response to *B. cinerea* infection [37]. It was also reported that PMEIs are involved in the plant’s responses to abiotic stresses; however, so far this involvement is underexplored [17]. The most highly upregulated defense-related genes were CYP81F2, PAD3, and WRKY40; all were upregulated more than 40-fold in each AnPME line (Figure 5c). PAD3 is induced in plants under fungal pathogenesis and is involved in camalexin biosynthesis [83]. CYP81F2 is involved in glucosinolate biosynthesis in response to fungal pathogenesis as well [84]. The WRKY family of transcription factors is involved in broad, complex interactions in defense response [85]. WRKY40 in particular regulates mitogen-activated protein kinase cascades and is implicated in CWI in Arabidopsis [57,58,86,87,88]. Other genes significantly upregulated in AnPME plants include β-G2, JR1, PR1, PR5, and WR3 (Figure 5c). Upregulation of β-G2 in Arabidopsis inhibits fungal growth [89,90]. PR1 and PR5 are both defense proteins induced under pathogenesis and are involved in salicylic acid metabolism and systemic acquired resistance [91,92]. JR1 and WR3 are both induced upon wounding and are associated with hypersensitive-like cell death [93]. The genes upregulated in AnPME plants are important antimicrobial defenses, including SA- and JA-responsive elements. SA and JA signaling are generally perceived as antagonistic mechanisms. However, when SA and JA are present in low concentrations, SA and JA pathways can also work synergistically to increase expression in both pathways [94]. This suggests a defense priming of both SA and JA pathways in AnPME plants in preparation for pathogenesis. It is unclear at this point whether AnPME plants are initiating defense responses as a result of cell wall modifications, or whether defenses are triggered by directly sensing the presence of a microbial CWDE such as *A. nidulans* PME. It is also unclear whether growth inhibition is due solely to Ca^2+^-mediated crosslinking of pectate, defense response activation, or whether both processes contribute to a degree. In addition, we do not know whether defense responses are affected in AnPME plants infected with *B. cinerea*. Additional studies in the future will be required to clarify the impact of microbial PME constitutively expressed in the apoplast on plant responses to biotic stress.

## 4. Materials and Methods

### 4.1. Transgene Construct

cDNA encoding *A. nidulans* AnPME (AN3390) was amplified from *Pichia pastoris* recombinant strains [95] from the Fungal Genetics Stock Center. The sequence was amplified by PCR with primers containing restriction sites KpnI and HindIII, respectively. After restriction digest, the AnPME fragment was ligated into a cassette containing (from 5′ to 3′) sequences encoding: an *Arabidopsis thaliana* β-expansin signal peptide, AnPME, and a green fluorescent protein marker (smGFP) fused to the C-terminus of AnPME. This expression cassette was subcloned into a pMLBart binary vector backbone with a Cauliflower Mosaic Virus 35S promoter driving expression of the cassette, as described in Fursova et al. [96]. Transformation was performed via *Agrobacterium tumefaciens*-mediated floral dip, and the resulting seed was selected to a homozygous T4 generation [97].

### 4.2. Plant Materials and Growth Conditions

*Arabidopsis* seeds were sterilized with sequential treatments of 70% ethanol and 0.5% bleach, washed with sterile water, and planted on 1/2-strength Murashige and Skoog medium (1/2MS) [98] with 2% sucrose and 0.3% Gelrite (Research Products International, Mt. Prospect, IL, USA). Plants were then transplanted into wet LC-1 potting soil mix (Sun Gro Horticulture, Agawam, MA, USA) 14 days after germination in a growth chamber with controlled conditions: 12-h light/12-h dark at 21 °C, with relative humidity of 65% and light intensity of 160 µmol s^−1^ m^−2^.

### 4.3. Cell Wall Extraction

Cell walls were isolated as described in Zabotina et al. [99]. 3 replicates of whole aerial parts of 10 plants were harvested and cut into 1-cm length segments. Tissue was frozen in liquid N_2_ and ground into a fine powder with a mortar and pestle. After homogenization, tissues were incubated in 80% ethanol at 80 °C twice for 1 h, and further homogenized with a PolyTron (Kinematica, Inc., Bohemia, NY, USA) at 15,000 rpm for 5 min. Cell walls were collected by centrifugation at 12,000× *g* and washed with 80% ethanol followed by several washes with 100% acetone until the supernatant turned clear. The cell walls were incubated in a solution of 20% SDS with 5 mM sodium metabisulfite at 4 °C for 16 h and washed five times with distilled water. Finally, the extract was incubated in 1:1 chloroform:methanol solution at room temperature for 20 min, washed three times with 100% acetone, and air-dried at 50 °C.

### 4.4. Cell Wall Saccharification Assays

For the digestion of pectins, 3 replicates of 5 mg of dry cell wall material were incubated with a mixture of 50 units of endo-polygalacturonase (Megazyme International, Wicklow, Ireland) and 15 units of PME (PROZOMIX LTD, Haltwhistle, UK) in a 0.3-mL total volume of sodium phosphate buffer (pH 6.0) for 24 h at 37 °C. Saccharification assays were performed as described in Pogorelko et al. (2011) with some modifications. Cell walls from 3 replicates of 10 4-week old plants (5 mg of fresh tissue) were incubated in 0.1 mL of citrate buffer (pH 4.9) containing 4 units of cellulase (from *Trichoderma reesei*, Sigma–Aldrich, C62730) and 1 unit of cellobiase (from *A. niger*, Sigma–Aldrich, C6105) on the shaker at 37 °C. The reaction was terminated by heating at 100 °C for 15 min. Supernatants were collected by centrifugation at 10,000× *g*, and the amount of reducing sugars released was analyzed by p-hydroxybenzoic acid hydrazide (PAHBAH) assay.

### 4.5. PAHBAH Assay of Reducing Sugars

Reducing sugars were measured from cell walls of 3 replicates of 10 pooled plants each using the PAHBAH assay [100] with minor modifications. Briefly, 15 µL of supernatant from each enzyme assay was mixed with 135 mL of freshly prepared PAHBAH reagent (1 volume of 5% p-hydroxybenzoic acid hydrazide in 5% HCl mixed with 9 volumes of 1.25% trisodium citrate, 0.11% calcium chloride, and 2% sodium hydroxide) and heated at 95 °C for exactly 6 min. Absorbance was measured at 410 nm using a microplate reader (BioTek Instruments, Inc., Winooski, VT, USA). Calculations were done using a standard curve prepared using different concentrations of glucose.

### 4.6. Cell Wall Monosaccharide Analysis

To determine monosaccharide composition, 3 replicates of cell walls from 10 plants were used. 1 mg of dry de-starched cell wall was hydrolyzed with 2 N trifluoroacetic acid at 120 °C for 2 h. The hydrolysates were dried at 50 °C, re-dissolved in water, and analyzed by high-performance anion-exchange chromatography with pulsed-amperometric detection using a CarboPac PA-20 column (3 mm × 150 mm; Dionex, Sunnyvale, CA, USA) as described earlier [99]. Monosaccharides were separated using a gradient of 100 mM NaOH in water at 0.5 mL min^−1^ under the following conditions: 0–0.05 min—12 mM NaOH; 0.05–26 min—0.65 mM NaOH; 26–46 min—300 mM NaOH; 46–55 min—12 mM NaOH. Monosaccharide standards included L-Fuc, L-Rha, L-Ara, D-Gal, D-Glc, D-Xyl, D Man, D-GalA, and D-GlcA (all from Sigma–Aldrich, St. Louis, MO, USA). To determine response factors, standard curves were created using mixtures of all standard monosaccharides at different concentrations.

### 4.7. Methylester Content Determination

Methylester content was determined by a method adapted from Klavons and Bennett [101]. Three replicates of 5 mg of total cell wall were saponified in 1 M NaOH for 24 h, then centrifuged at 12,000× *g* for 10 min. The supernatant was transferred to a new 1.5 mL tube, centrifuged again, transferred to another tube to remove any particulates, then neutralized with 1 M HCl. 50 μL of the supernatant was added to 50 μL of alcohol oxidase (0.6 U mL^−1^) in 0.1 M sodium phosphate buffer pH 7.5, then incubated on a shaker for 15 min at 25 °C. Then, 100 μL of a solution containing 0.02 M 2,4-pentanedione, 2 M ammonium acetate, and 0.05 M acetic acid in sodium phosphate buffer pH 7.5 was added to the reaction. After 10 min of incubation at 68 °C, samples were cooled on ice and centrifuged. The solution was pipetted into a 96-well plate, then quantified in a spectrophotometer at 412 nm wavelength and compared to a standard curve of methanol concentrations to determine molar concentration.

### 4.8. Cell Length Measurements

Six one-week-old plants were incubated in a solution of 10 μg mL^−1^ propidium iodide in distilled water for one minute, then washed briefly by soaking in distilled water three times for one minute per wash. Roots were then mounted on a microscope slide with a cover slip and imaged using a Leica SP5 confocal microscope at 535 nm excitation and 617 nm emission wavelengths (Iowa State University Confocal and Multiphoton Facility). Cell length was then measured using ImageJ image processing software.

### 4.9. Salt and Osmotic Stress Assays

Plants grown for salt stress were germinated on 1/2MS medium plates grown vertically for 5 days until cotyledons had fully expanded. After 5 days of growth, seedlings were transplanted to new plates containing either 1/2MS with no stress, 1/2MS medium containing 100 mM NaCl (salt stress), and 1/2MS containing 300 mM mannitol (osmotic stress). Root lengths of ≥75 plants were imaged and measured using ImageJ software, then plants were grown vertically for 5 days, at which point root length was measured again to determine the growth rate for each seedling.

### 4.10. Determination of Reactive Oxygen Species (ROS) Accumulation

Rosette leaves were carefully detached from 12 4-week old plants and incubated in a solution of 1 mg mL^−1^ 3,3′-diaminobenzene, 0.05% Tween-20, and 10 mM Na_2_HPO_4_, pH 3.0 for 24 h. Leaves were then transferred to a bleaching solution containing 3:1:1 ethanol: acetic acid: glycerol and heated to 95 °C for 15 min. After cooling, leaves were rinsed twice in fresh bleaching solution and imaged immediately.

### 4.11. Botrytis cinerea Preparation and Inoculation

*B. cinerea* was grown for 15 days on potato dextrose agar at 23 °C under a 12-h photoperiod before collecting spores. Spores were collected by washing the plates with 5 mL sterile water, then filtered through glass wool to remove mycelia. Conidia were diluted to a stock concentration of 1 × 10^6^ conidia mL^−1^. Conidia were then further diluted to a working concentration of 1 × 10^5^ conidia mL^−1^ in 25% grape juice, then 10 μL of conidia solution was transferred to 12 detached Arabidopsis leaves in agar plates. The inoculated leaves were grown in a growth chamber as described above for 48 h, and then lesion area measurements were taken using ImageJ software.

### 4.12. RNA Extraction, cDNA Synthesis, and Real-Time Quantitative PCR

Total RNA from 5 3-week old plants grown on 1/2-strength MS medium was extracted with Trizol-chloroform (Invitrogen). After treatment with DNase, RNA was converted to cDNA using the SuperScript III First Strand Synthesis system (Invitrogen). Quantification of gene expression was performed using the Maxima SYBR Green qPCR Master Mix (2X; Thermo Scientific, Waltham, MA, USA) and the CFX-96 thermal cycler (Bio-Rad) normalized against ACTIN2 as an internal control. Relative gene expression was calculated using the comparative threshold cycle method [102]. All primers used in this study are attached in Table S1.

### 4.13. Stomatal Measurements

Seeds were sterilized in 30% bleach with 0.1% SDS, rinsed with sterile water, and resuspended in 0.15% agar. After stratification at 4 °C for 3 days, seeds were grown on ½ MS (2.2 g/L Murashige and Skoog salts, 0.6 g/L MES, pH 5.6), 1% (*w/v*) sucrose 0.8%, agar plates for 6 days. Healthy seedlings were picked to float on stomatal function assay solutions (dark-induced closure: 20 mM KCl, 1 mM CaCl_2_, and 5 mM MES- KOH, pH 6.15; light-induced opening: 50 mM KCl, 0.1 mM CaCl_2_, and 10 mM MES-KOH, pH 6.15). To induce opening/closure of stomata, samples were pretreated under dark/light, respectively, for 2.5 h. Seedlings were then transferred to light/dark environments, respectively, and imaged. Guard cell images were recorded using a Nikon D5100 DSLR camera and Zeiss Cell Observer SD microscope before and after induction; guard cell dimensions were measured using ImageJ.

## 5. Conclusions

In this study, we show that pectin methylester status occupies an intersection between cell wall mechanics and plant stress response. In Arabidopsis, expression of AnPME modifies cell wall HG, which in turn inhibits plant growth and activates plant stress responses. Reduction of pectin methylesterification increases cell wall stiffness and limits cell elongation, promotes pectin degradation, and can positively affect plant resistance to high salt and mannitol concentrations. Resistance to high salt and mannitol concentrations could be achieved by free pectic carboxyl groups chelating Na^+^ ions or retaining more water. Despite these changes in cell wall composition and recalcitrance, AnPME plants displayed robust defense responses. The observations reported here show that HG methylesterification status is a critical factor in plant growth and protection. It is clear that HG is important for plant growth and defense. In order to determine whether AnPME-expressing plants are initiating defenses in response to either cell wall modification or the presence of AnPME itself, a catalytically inactive AnPME will be produced, transformed into Arabidopsis plants, and assayed for the same phenotypes in following studies. In the future, a comparison of plants expressing active and inactive AnPME enzymes could reveal the exact relationship between pectin methylesterification, cell wall integrity, and defense responses.

The cell wall is an indispensable component of plant life. Its ability to control growth and to respond to changes around it are just two of the cell wall’s many functions. Here, we have shown that pectin status directly affects growth and defense in Arabidopsis plants. Thus, this study advances our understanding of the contribution of cell wall components to the numerous functions of the cell wall, including the growth-defense tradeoff shown here.

## Supplementary Materials

The following are available online at https://www.mdpi.com/2223-7747/9/11/1558/s1, Figure S1: Comparison of root lengths under optimal and osmotic-stressed conditions, Table S1: Primers used for RT-PCR.
